# Supplementary material for: Quantity and Distribution of Muscle Spindles in Animal and Human Muscles
Source: Int J Mol Sci. 2024 Jul 3;25(13):7320. doi: 10.3390/ijms25137320 (PMC11242712; doi:10.3390/ijms25137320)
Supplement: Supplementary file 1 [file ijms-25-07320-s001.zip › ijms-3024076-supplementary/Supplementary Figure S1A-G.pdf]

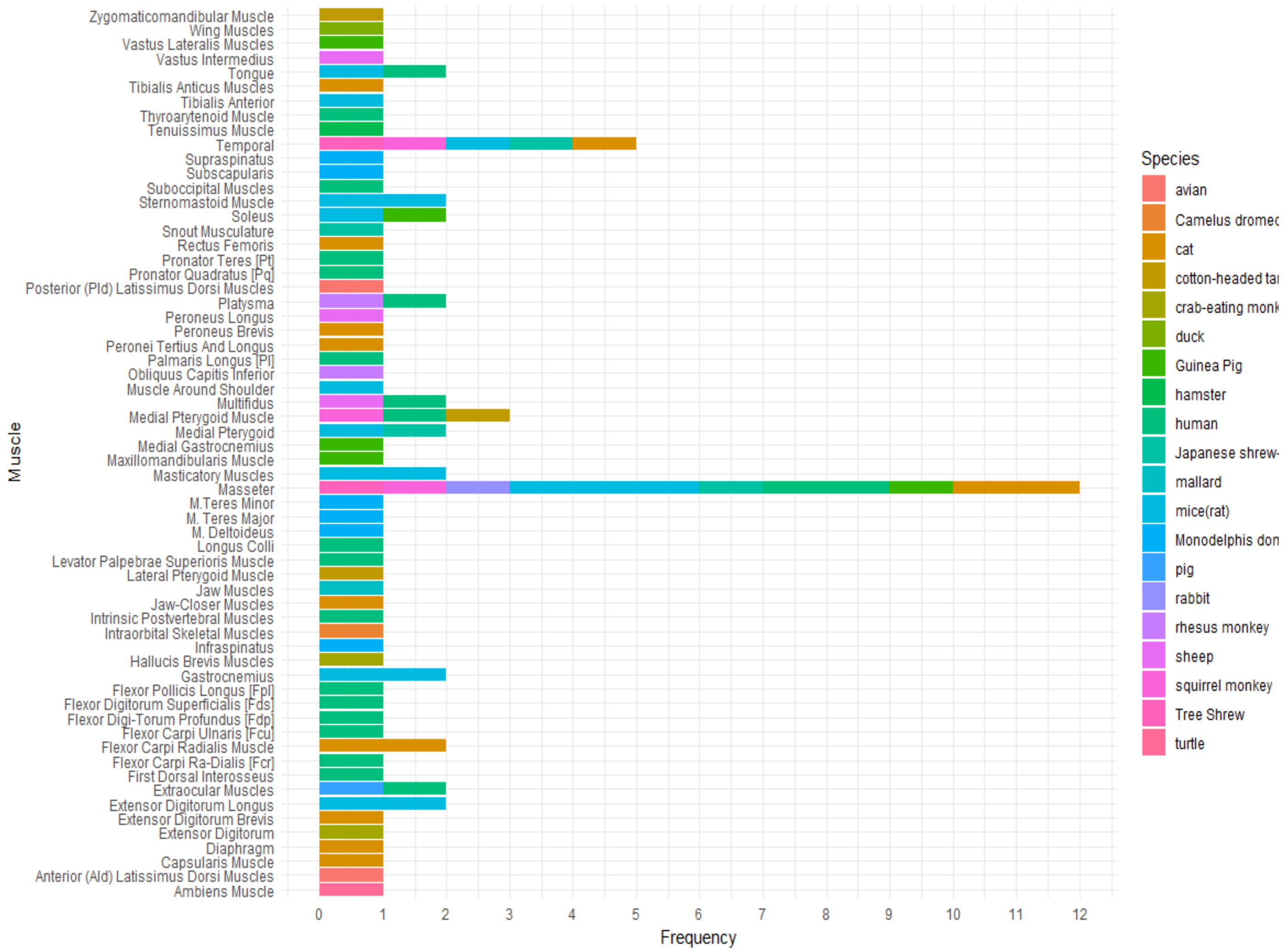
Zygomaticomandibular Muscle
Wing Muscles
Vastus Lateralis Muscles
Vastus Intermedius
Tongue
Tibialis Anticus Muscles
Tibialis Anterior
Thyroarytenoid Muscle
Tenuissimus Muscle
Temporal
Supraspinatus
Subscapularis
Suboccipital Muscles
Sternomastoid Muscle
Soleus
Snout Musculature
Rectus Femoris
Pronator Teres [Pt]
Pronator Quadratus [Pq]
Posterior (Pld) Latissimus Dorsi Muscles
Platysma
Peroneus Longus
Peroneus Brevis
Peronei Tertius And Longus
Palmaris Longus [Pl]
Obliquus Capitis Inferior
Muscle Around Shoulder
Multifidus
Medial Pterygoid Muscle
Medial Pterygoid
Medial Gastrocnemius
Maxillomandibularis Muscle
Masticatory Muscles
Masseter
M.Teres Minor
M. Teres Major
M. Deltoideus
Longus Colli
Levator Palpebrae Superioris Muscle
Lateral Pterygoid Muscle
Jaw Muscles
Jaw-Closer Muscles
Intrinsic Postvertebral Muscles
Intraorbital Skeletal Muscles
Infraspinatus
Hallucis Brevis Muscles
Gastrocnemius
Flexor Pollicis Longus [Fpl]
Flexor Digitorum Superficialis [Fds]
Flexor Digi-Torum Profundus [Fdp]
Flexor Carpi Ulnaris [Fcu]
Flexor Carpi Radialis Muscle
Flexor Carpi Ra-Dialis [Fcr]
First Dorsal Interosseus
Extraocular Muscles
Extensor Digitorum Longus
Extensor Digitorum Brevis
Extensor Digitorum
Diaphragm
Capsularis Muscle
Anterior (Ald) Latissimus Dorsi Muscles
Ambiens Muscle
Muscle
0 1 2 3 4 5 6 7 8 9 10 11 12
Frequency
Species
avian
Camelus dromec
cat
cotton-headed tai
crab-eating monk
duck
Guinea Pig
hamster
human
Japanese shrew-
mallard
mice(rat)
Monodelphis don
pig
rabbit
rhesus monkey
sheep
squirrel monkey
Tree Shrew
turtle

(**A**)

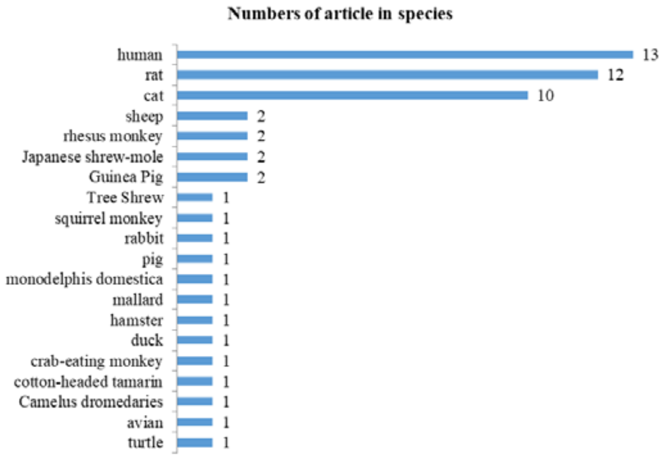
Numbers of article in species
human 13
rat 12
cat 10
sheep 2
rhesus monkey 2
Japanese shrew-mole 2
Guinea Pig 2
Tree Shrew 1
squirrel monkey 1
rabbit 1
pig 1
monodelphis domestica 1
mallard 1
hamster 1
duck 1
crab-eating monkey 1
cotton-headed tamarin 1
Camelus dromedaries 1
avian 1
turtle 1

(**B**)

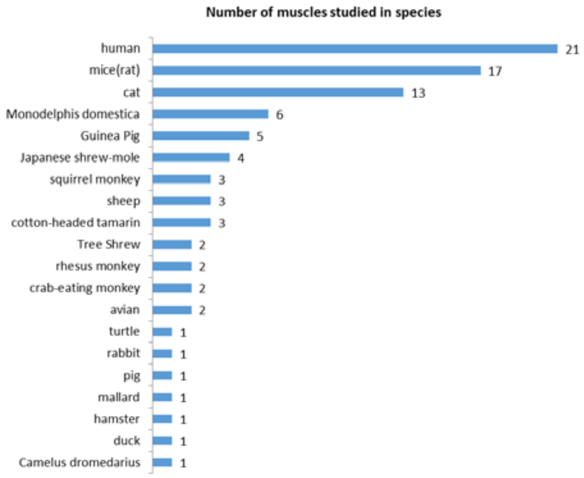
Number of muscles studied in species
human 21
mice(rat) 17
cat 13
Monodelphis domestica 6
Guinea Pig 5
Japanese shrew-mole 4
squirrel monkey 3
sheep 3
cotton-headed tamarin 3
Tree Shrew 2
rhesus monkey 2
crab-eating monkey 2
avian 2
turtle 1
rabbit 1
pig 1
mallard 1
hamster 1
duck 1
Camelus dromedarius 1

(**C**)

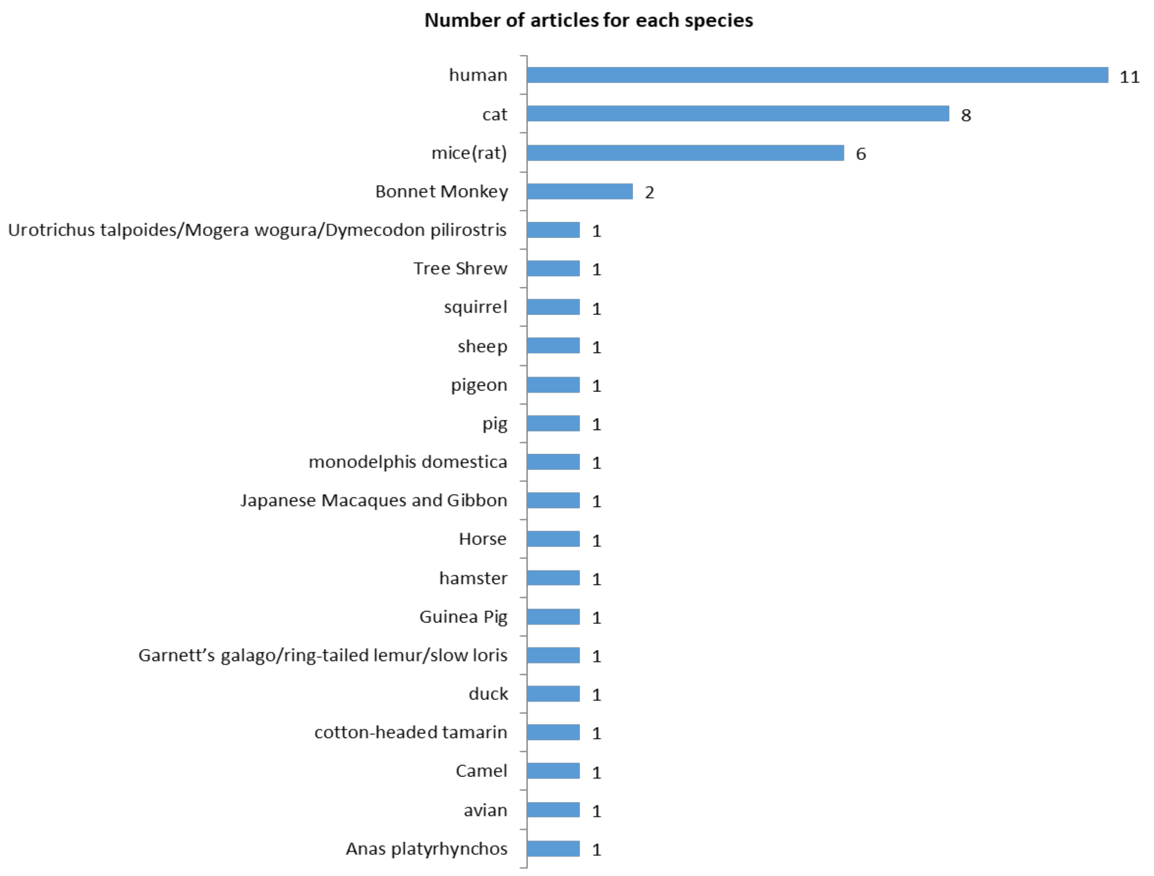

Number of articles for each species
human
11
cat
8
mice(rat)
6
Bonnet Monkey
2
Urotrichus talpoides/Mogera wogura/Dymecodon pilirostris
1
Tree Shrew
1
squirrel
1
sheep
1
pigeon
1
pig
1
monodelphis domestica
1
Japanese Macaques and Gibbon
1
Horse
1
hamster
1
Guinea Pig
1
Garnett's galago/ring-tailed lemur/slow loris
1
duck
1
cotton-headed tamarin
1
Camel
1
avian
1
Anas platyrhynchos
1

(**D**)

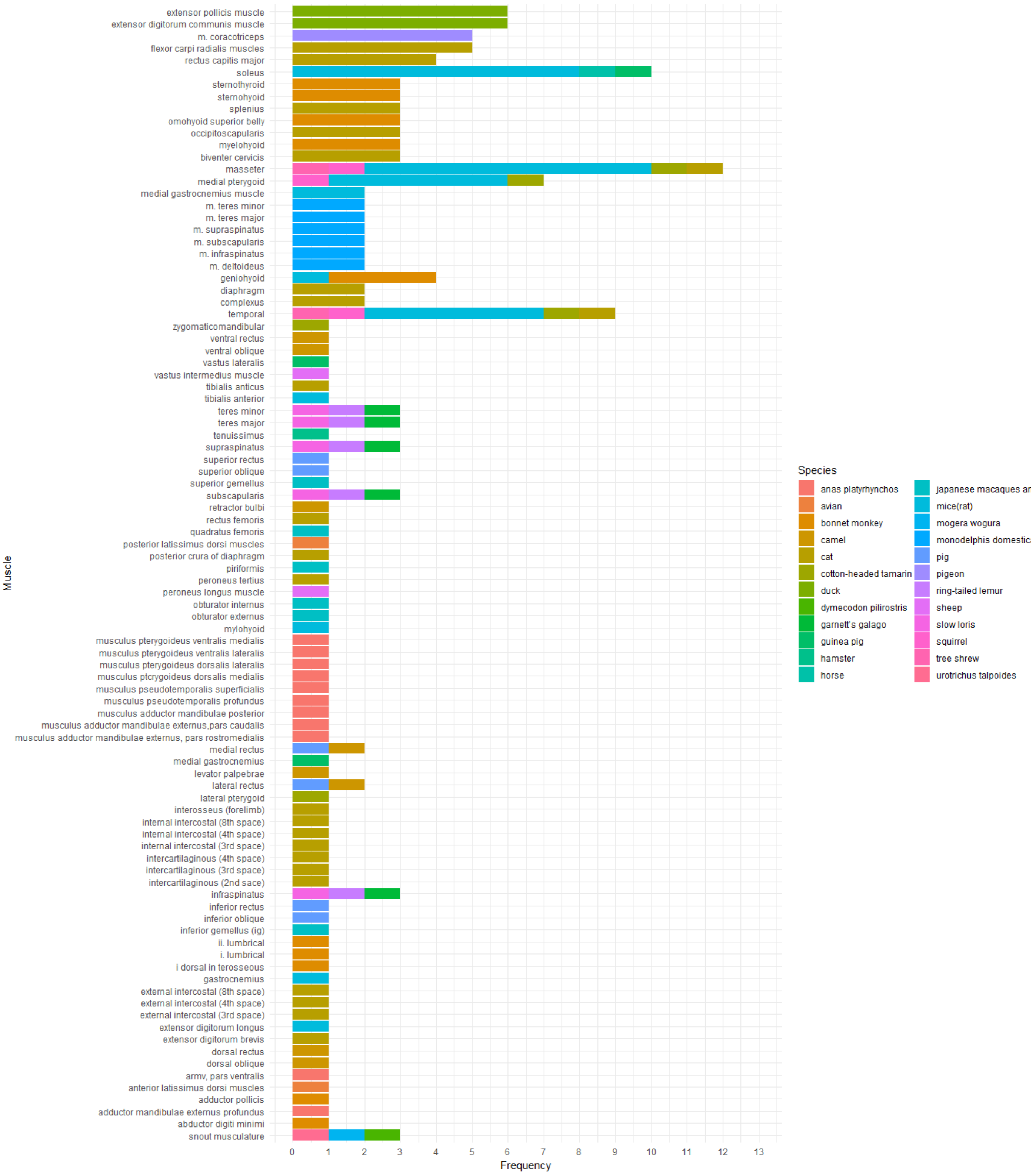

extensor pollicis muscle
extensor digitorum communis muscle
m. coracotriceps
flexor carpi radialis muscles
rectus capitis major
soleus
sternothyroid
sternohyoid
splenius
omohyoid superior belly
occipitoscapularis
myelohyoid
biventer cervicis
masseter
medial pterygoid
medial gastrocnemius muscle
m. teres minor
m. teres major
m. supraspinatus
m. subscapularis
m. infraspinatus
m. deltoideus
geniohyoid
diaphragm
complexus
temporal
zygomaticomandibular
ventral rectus
ventral oblique
vastus lateralis
vastus intermedius muscle
tibialis anticus
tibialis anterior
teres minor
teres major
tenuissimus
supraspinatus
superior rectus
superior oblique
superior gemellus
subscapularis
retractor bulbi
rectus femoris
quadratus femoris
posterior latissimus dorsi muscles
posterior crura of diaphragm
piriformis
peroneus tertius
peroneus longus muscle
obturator internus
obturator externus
mylohyoid
musculus pterygoideus ventralis medialis
musculus pterygoideus ventralis lateralis
musculus pterygoideus dorsalis lateralis
musculus ptcrygoideus dorsalis medialis
musculus pseudotemporalis superficialis
musculus pseudotemporalis profundus
musculus adductor mandibulae posterior
musculus adductor mandibulae externus,pars caudalis
musculus adductor mandibulae externus, pars rostromedialis
medial rectus
medial gastrocnemius
levator palpebrae
lateral rectus
lateral pterygoid
interosseus (forelimb)
internal intercostal (8th space)
internal intercostal (4th space)
internal intercostal (3rd space)
intercartilaginous (4th space)
intercartilaginous (3rd space)
intercartilaginous (2nd sace)
infraspinatus
inferior rectus
inferior oblique
inferior gemellus (ig)
ii. lumbrical
i. lumbrical
i dorsal in terosseous
gastrocnemius
external intercostal (8th space)
external intercostal (4th space)
external intercostal (3rd space)
extensor digitorum longus
extensor digitorum brevis
dorsal rectus
dorsal oblique
amv, pars ventralis
anterior latissimus dorsi muscles
adductor pollicis
adductor mandibulae externus profundus
abductor digiti minimi
snout musculature
Muscle
0 1 2 3 4 5 6 7 8 9 10 11 12 13
Frequency
Species
anas platyrhynchos
avian
bonnet monkey
camel
cat
cotton-headed tamarin
duck
dymecodon pilirostris
garnett's galago
guinea pig
hamster
horse
japanese macaques ar
mice(rat)
mogera wogura
monodelphis domestic
pig
pigeon
ring-tailed lemur
sheep
slow loris
squirrel
tree shrew
urotrichus talpoides

(E)

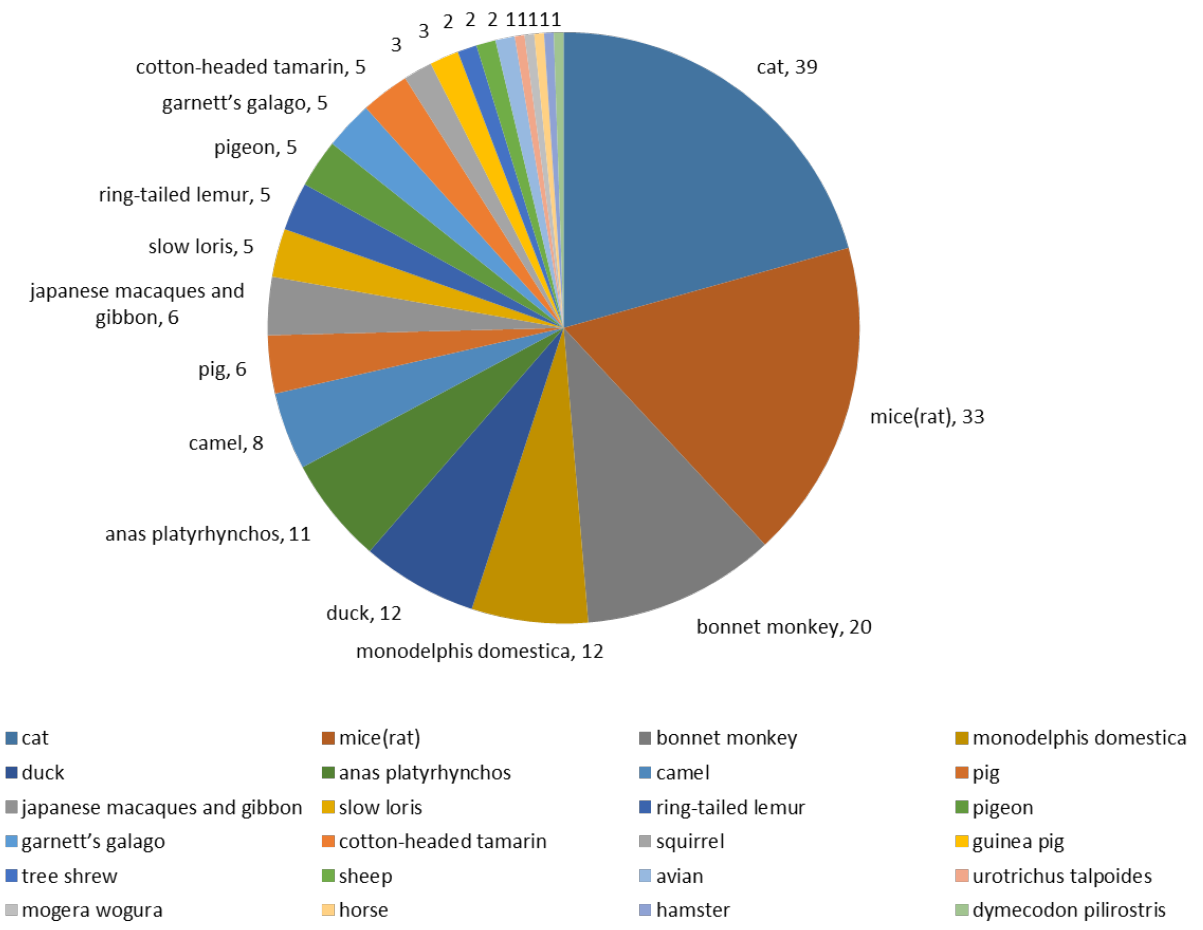

cat, 39
mice(rat), 33
bonnet monkey, 20
monodelphis domestica, 12
duck, 12
anas platyrhynchos, 11
camel, 8
pig, 6
japanese macaques and gibbon, 6
slow loris, 5
ring-tailed lemur, 5
pigeon, 5
garnett's galago, 5
cotton-headed tamarin, 5
3
3
2
2
2
11111
cat
mice(rat)
bonnet monkey
monodelphis domestica
duck
anas platyrhynchos
camel
pig
japanese macaques and gibbon
slow loris
ring-tailed lemur
pigeon
garnett's galago
cotton-headed tamarin
squirrel
guinea pig
tree shrew
sheep
avian
urotrichus talpoides
mogera wogura
horse
hamster
dymecodon pilirostris

(**F**)

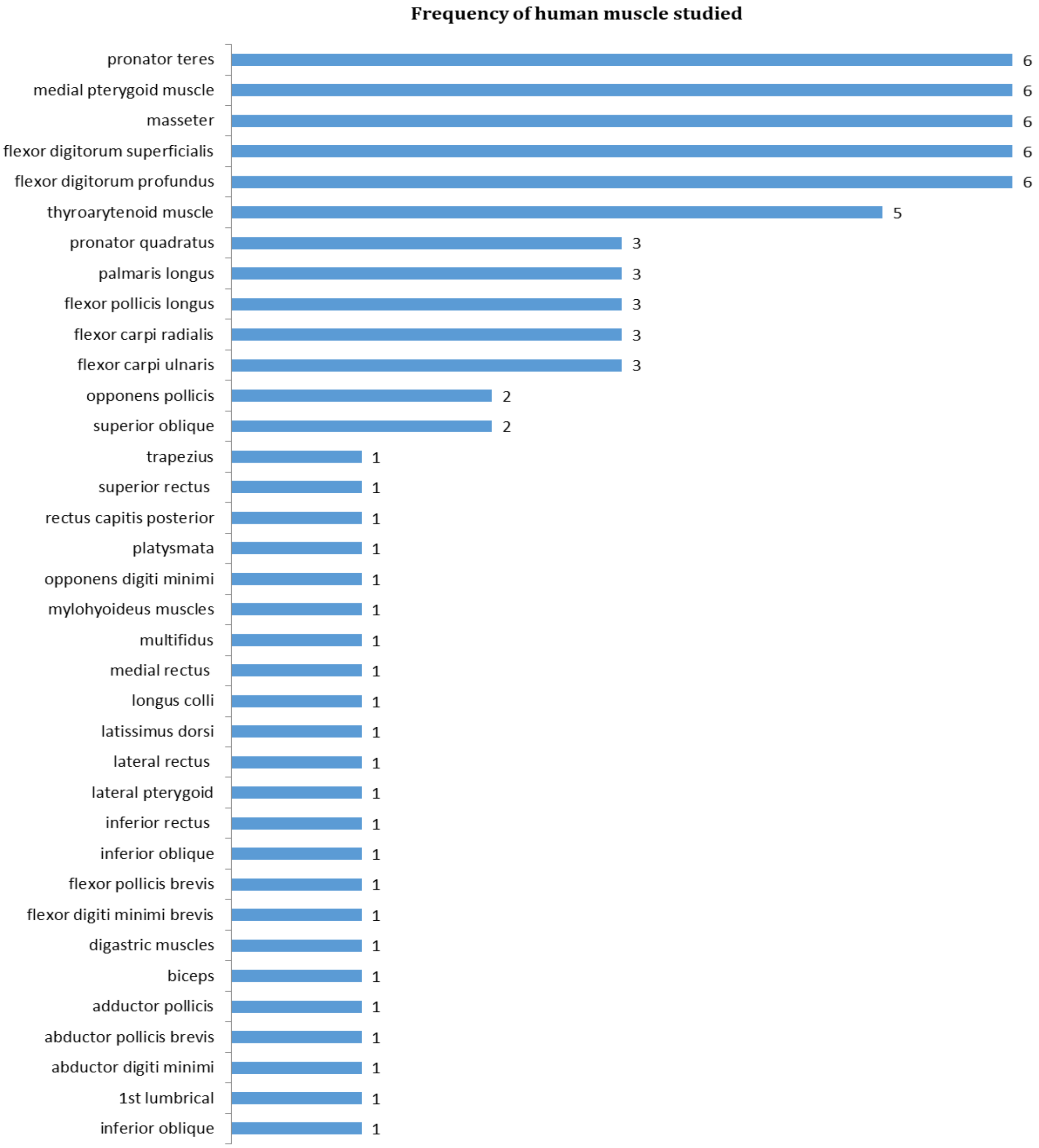

(**G**)

Figure S1. Literature review results. **A:** Results of part 2.1, distribution of MSs, frequency plots of different muscles analyzed in different species. Masseter was the most frequently evaluated muscle while the ambiens muscle and the other 48 kind muscles were the lowest (1 time). The bars stand for the frequency of investigated muscle, and species were presented in different color. **B:** Results of part 2.1, distribution of MSs, numbers of articles which focus on each species. Humans, rodents and cats were the most popular objects in MS research. **C:** Results of part 2.1, distribution of MSs, number of studied muscles in each species, total 90 muscles. **D:** Results of part 2.2, numbers of MSs, a summary of how many articles in each species, one article owned 1or more species usually. **E:** Results of 2.2 numbers of MSs, frequency of each muscle in animal were presented. **F:** Related to E, amounts of muscles studied in each animal species. **G:** Results of 2.2 numbers of MSs, the frequency of studied muscles in human were presented.
