# Supplementary material for: Quantity and Distribution of Muscle Spindles in Animal and Human Muscles
Source: Int J Mol Sci. 2024 Jul 3;25(13):7320. doi: 10.3390/ijms25137320 (PMC11242712; doi:10.3390/ijms25137320)
Supplement: Supplementary file 1 [file ijms-25-07320-s001.zip › ijms-3024076-supplementary/supplementary Tables/Supplementary table S1.pdf]

Supplementary Table S1 Outstanding work of MSs Distribution

Muscle	Close to IMCT/Fascia	Close to nerve(NEPs)	Close to vessels	Superficial	Deep	Proximal	Middle	Distal	High density area	Species	Method	References
tibialis anterior, extensor digitorum longus, gastrocnemius, soleus muscle		★(extensor digitorum longus, soleus muscle				★	★			mice	H&E	(Lian et al. 2022)[31]
multifidus muscle	★						★			sheep	Van Gieson’s	(James et al. 2022)[40]
masseter muscle					★				deep layer of the posterior superficial region	mice	immunohistochemistry	(Iwao SATO. 2007)[50]
Intraorbital Skeletal Muscles		★		★						Camelus dromedarius	Van Gieson’s	(Abuel-Atta, DeSantis, and Wong 1997)[30]
wing muscles						★	★			duck	Delafield's H&E	(Adal and Chew Cheng 1980)[51]

intrinsic postvertebral muscles	★							human	Van Gieson's stain with Weigert's haematoxylin	(Amonoo-Kuofi 1982)[52]
Muscle around shoulder						★	Muscle-tendon junction	mice	$AgNO_3$	(Backenköhler, Halata, and Strasmann 1996)[53]
sternomastoid muscle		★					ventral portion	rat	ATPase	(Giuriati et al. 2018)[39]
platysma		★		★	★		cranial two-thirds area	human	H&E	(May et al. 2018)[44]
platysma				★			regions connected to the cheek pouch	rhesus monkey	H&E	(May et al. 2022)[45]
Anterior (ALD) and posterior (PLD) latissimus dorsi muscles	★(branch)		★(PDL)	★	★		Slow muscle fiber: well-distributed; Fast fiber: NEPs	avian	H&E	(Ovalle, Dow, and Nahirney 1999)[54]
gastrocnemius				★	★		longitudinal axis of the medial gastrocnemius	rat	Masson-Goldner trichome method	(Piotr et al. 2023)[55]

vastus intermedius, peroneus longus				★ (peroneus longus)	★ (peroneus longus)	★(vastus intermedius)		sheep	periodic acid Shiff reaction and Mayer's hematoxylin	(Watanabe and Suzuki 1999)[56]
rectus femoris, tibialis anticus muscles	★				★			cat	H&E	(Barker and Chin 1960)[57]
medial pterygoid muscle			★		★		Middle area	human	Weigert-Van Gieson	(Bhojwani et al. 2017)[58]
Extraocular Muscles		★		★	★	★	Entire muscle length	pig	Azan and ferric oxide	(Blumer et al. 2001)[59]
Jaw Muscles							Non specific	mallard	H&E	(Bout and Dubbeldam 1991)[49]
longus colli		★ anterolateral						human	Masson’s trichrome	(Boyd-Clark, Briggs, and Galea 2002)[60]
multifidus							closely to vertebral lamina	human	Masson’s trichrome	(Boyd-Clark et al. 2002)[60]
masseter			★	★				rabbit	Light-microscope	(Bredman, Weijs, and Brugman 1991)[61]

Extensor Digitorum Brevis			★			★	midway between the dorsal and ventral surfaces	cat	van Gieson	(Bridgman, Eldred, and Eldred 1962)[62]
jaw-closer muscles				★				cat	Van Gieson	(Burhanudin, McDonald, and Rowlerson 1996)[63]
Capsularis Muscle				★				cat	ATPase	(Eldred, Yung, and Roy 1997)[64]
masseter				★				human	ATPase	(Eriksson and Thornell 1987)[65]
flexor carpi radialis muscle				★			In slow fiber(deep area)	cat	ATPase	(Gonyea and Ericson 1977)[66]
ambiens muscle			★				In slow fiber	turtle	NA	(Hermanson, Lennard, and Takamoto 1986)[67]
extensor digitorum longus	★	★						mice	Myosin ATPase	(Johnson and Ovalle 1986)[34]
masseter							uneven	mice	Miller's, van Gieson's or Bodian's	(Karlsen 1965)[68]

masseter	★	★		★			Japanese shrew-mole	H&E	(Kubota and Masegi 1972b)[41]
Medial pterygoid	★	★	★	★		Anterolateral of muscle	Japanese shrew-mole	H&E	(Kubota and Masegi 1972b)[41]
Temporal muscle		★		★			Japanese shrew-mole	H&E	(Kubota and Masegi 1972b)[41]
Snout Musculature		★		★	★		Japanese shrew-mole	H&E, azan	(Kubota and Masegi 1972a)[69]
medial pterygoid muscle		★		★			cotton-headed tamarin	H&E	(Kubota and Masegi 1975)[70]
zygomaticomandi bular muscle				★			cotton-headed tamarin	H&E	(Kubota and Masegi 1975)[70]
lateral pterygoid muscle				★			cotton-headed tamarin	H&E	(Kubota and Masegi 1975)[70]
Temporal muscle				★		Anterior area	squirrel monkey	H&E	(Kubota, Masegi, and Osanai 1973)[71]

medial pterygoid muscle				★		★			squirrel monkey	H&E	(Kubota et al. 1973)[71]
masseter						★			squirrel monkey	H&E	(Kubota et al. 1973)[71]
Temporal muscle						★		Anterior area	Tree Shrew	H&E, Azan, silver impregnation, Weigert's myelin sheath	(Kubota, Masegi, and Quanbunchan 1974)[72]
masseter				★		★			Tree Shrew	H&E, Azan, silver impregnation, Weigert's myelin sheath	(Kubota et al. 1974)[72]
suboccipital muscles	★	★							human	Teased silver	(Kulkarni, Chandy, and Babu 2001)[35]
masticatory muscles				★		★	★		rat	Weigert's iron Haematoxylin, van Gieson	(Lennartsson 1980)[73]
Extraocular Muscles			★		★		★	periphery within a semicircular area	human	toluidine blue	(Lukas et al. 1994)[74]

Masseter, Temporal			★	★		★		cat	Holmes silver	(Lund et al. 1978)[42]
Subscapularis, supraspinatus,infr aspinatus							evenly	Monodelphis domestica	silver	(Maass, Baumann, and Halata 2001)[75]
M.teres minor				★		★	2/3 close to origin at the scapula	Monodelphis domestica	silver	(Maass et al. 2001)[75]
M. teres major				★			close to origin at the scapula	Monodelphis domestica	silver	(Maass et al. 2001)[75]
M. deltoideus		★		★(head)			lateral parts of the ventral, dorsal aspect	Monodelphis domestica	silver	(Maass et al. 2001)[75]
masseter, medial pterygoid,tempora lis	★						Located in Slow fiber	rat	H&E	(Maier 1979)[76]
soleus		★	★				evenly	Guinea Pig	trichrome stained	(Maier, Simpson, and Edgerton 1976)[77]
medial gastrocnemius, vastus lateralis		★	★		★		Located in Slow fiber	Guinea Pig	trichrome stained	(Maier et al. 1976)[77]

muscles											
Extensor Digitorum, Hallucis Brevis Muscles						★		Middle of muscle belly	crab-eating monkey	toluidine blue	(Matsumura and Saito 1997)[78]
masseter			★	★		★		muscle belly	Guinea Pig	H&E	(Odagiri, Kubota, and Shibanai 1993)[79]
maxillomandibularis muscle								medial part of the muscle	Guinea Pig	H&E	(Odagiri et al. 1993)[79]
masseter				★					human	mATPase	(Osterlund et al. 2011)[12]
tenuissimus muscle	★	★							hamster	H&E	(Patten and Ovalle 1992)[36]
pronator teres [PT]	★				★(INDR)		★(INDR)	INDR	human	Sihler's, H&E	(Zhou et al. 2023)[5]

flexor carpi ra-dialis [FCR]	★		★(INDR)			human	Sihler's, H&E	(Zhou et al. 2023)[5]
palmaris longus [PL]	★	★(INDR)				human	Sihler's, H&E	(Zhou et al. 2023)[5]
flexor carpi ulnaris [FCU]	★	★(INDR)				human	Sihler's, H&E	(Zhou et al. 2023)[5]
flexor digitorum superficialis [FDS]	★	★(INDR)	★(INDR)			human	Sihler's, H&E	(Zhou et al. 2023)[5]
flexor pollicis longus [FPL]	★			★(INDR)		human	Sihler's, H&E	(Zhou et al. 2023)[5]
flexor digi-torum profundus [FDP]	★	★(INDR)				human	Sihler's, H&E	(Zhou et al. 2023)[5]
pronator quadratus [PQ]	★		★(INDR)			human	Sihler's, H&E	(Zhou et al. 2023)[5]
sternomastoid muscle					Slow muscle fiber	rat	Azan and Goldner	(Zenker, Sandoz, and Neuhuber 1988)[80]

Levator Palpebrae Superioris Muscle				★				human	-	(Takahashi et al. 2021)[81]
Tongue	★							rat	ATPase	(Smith 1989)[82]
peronei tertius and longus	★			★				cat	Silver	(Scott and Young 1987)[83]
peroneus brevis	★			★	★	★		cat	Silver	(Scott and Young 1987)[83]
masseter			★			★		cat	ATPase	(Sciote 1993)[43]
thyroarytenoid muscle		★		★			superior medial quadrant	human	H&E	(Sanders et al. 1998)[84]
Tongue				★			posterior area near the base of the tongue	human	H&E	(Saigusa et al. 2004)[85]
First Dorsal Interosseus		★		★		★		human	deCastro's silver	(Sahinen and Kennedy 1972)[86]

flexor carpi radialis muscle	★	★	★	★		cat	Holmes	(Richmond and Stuart 1985)[87]
Obliquus capitis inferior	★				Slow muscle fiber	rhesus monkeys	ATPase,H&E	(Richmond, Singh, and Corneil 1999)[88]
Diaphragm	★				vertebral part of the muscle	cat	H&E	(Duron, Jung-Caillol, and Marlot 1978)[89]
Masticatory muscles					Slow muscle fiber	albino Wistar rats	ATPase	(Rokx, van Willigen, and Jansen 1984)[90]

★: Conclusion of the references; H&E: hematoxylin and eosin; IVD: intervertebral disc; NEPs: nerve-entry points; INDR: intramuscular nerve-dense region; IMCT: intramuscular connective tissue.

References

50. Sato, I.; Imura, K.; Miwa, Y.; Ide, Y.; Murata, M.; Sunohara, M. Distribution of Slow Muscle Fiber of Muscle Spindle in Postnatal Rat Masseter Muscle. Okajimas Folia Anat. Jpn. 2007, 84, 99–105. https://doi.org/10.2535/ofaj.84.99.
51. Adal, M.N.; Cheng, S.B.C. The Number, Distribution and Density of Muscle Spindles in Two Wing Muscles of the Domestic Duck. J. Anat. 1980, 131 Pt 3, 541–548.
52. Amonoo-Kuofi, H.S. The Number and Distribution of Muscle Spindles in Human Intrinsic Postvertebral Muscles. J. Anat. 1982, 135 Pt 3, 585–599.
53. Backenköhler, U.; Halata, Z.; Strasmann, T.J. The Sensory Innervation of the Shoulder Joint of the Mouse. Ann. Anat. Anat. Anz. Off. Organ. Anat. Ges. 1996, 178, 173–181. https://doi.org/10.1016/S0940-9602(96)80040-9.
54. Ovalle, W.K.; Dow, P.R.; Nahirney, P.C. Structure, Distribution and Innervation of Muscle Spindles in Avian Fast and Slow Skeletal Muscle. J. Anat. 1999, 194 Pt 3, 381–394. https://doi.org/10.1046/j.1469-7580.1999.19430381.x.
55. Piotr, M.; Skieresz‐Szewczyk, K.; Jackowiak, H.; Celichowski, J. Distribution and Length of Muscle Spindles and Their 3D Visualisation in the Medial Gastrocnemius of Male and Female Rats. J. Anat. 2023, 243, 658‐663. https://doi.org/10.1111/joa.13895.
56. Watanabe, K.; Suzuki, A. Distribution, Density, and Structure of Muscle Spindles in the Vastus Intermedius and the Peroneus Longus Muscles of Sheep. Okajimas Folia Anat. Jpn. 1999, 76, 203–219. https://doi.org/10.2535/ofaj1936.76.5_203.

57. Barker, D.; Chin, N.K. The Number and Distribution of Muscle-Spindles in Certain Muscles of the Cat. J. Anat. 1960, 94 Pt 4, 473–486.
58. Bhojwani, V.; Ghabriel, M.N.; Mihailidis, S.; Townsend, G.C. The Human Medial Pterygoid Muscle: Attachments and Distribution of Muscle Spindles. Clin. Anat. 2017, 30, 1064–1071. https://doi.org/10.1002/ca.22947.
59. Blumer, R.; Wasicky, R.; Brugger, P.C.; Hoetzenecker, W.; Wicke, W.L.; Lukas, J.R. Number, Distribution, and Morphologic Particularities of Encapsulated Proprioceptors in Pig Extraocular Muscles. Investig. Ophthalmol. Vis. Sci. 2001, 42, 3085–3094.
60. Boyd-Clark, L.C.; Briggs, C.A.; Galea, M.P. Muscle Spindle Distribution, Morphology, and Density in Longus Colli and Multifidus Muscles of the Cervical Spine. Spine 2002, 27, 694–701. https://doi.org/10.1097/00007632-200204010-00005.
61. Bredman, J.J.; Weijs, W.A.; Brugman, P. Relationships between Spindle Density, Muscle Architecture and Fibre Type Composition in Different Parts of the Rabbit Masseter. Eur. J. Morphol. 1991, 29, 297–307.
62. Bridgman, C.F.; Eldred, E.; Eldred, B. Distribution and Structure of Muscle Spindles in the Extensor Digitorum Brevis of the Cat. Anat. Rec. 1962, 143, 219–227. https://doi.org/10.1002/ar.1091430306.
63. Burhanudin, R.; McDonald, F.; Rowlerson, A. Muscle Spindles in the Jaw-Closer Muscles of the Domestic Cat. J. Anat. 1996, 188 Pt 2, 299–309.
64. Eldred, E.; Yung, L.; Roy, R.R. Spindle Representation Relative to Distribution of Muscle Fiber Types in the Cat Capsularis Muscle. Acta Anat. 1997, 159, 114–126. https://doi.org/10.1159/000147974.
65. Eriksson, P.O.; Thornell, L.E. Relation to Extrafusal Fibre-Type Composition in Muscle-Spindle Structure and Location in the Human Masseter Muscle. Arch. Oral. Biol. 1987, 32, 483–491. https://doi.org/10.1016/s0003-9969(87)80009-2.
66. Gonyea, W.J.; Ericson, G.C. Morphological and Histochemical Organization of the Flexor Carpi Radialis Muscle in the Cat. Am. J. Anat. 1977, 148, 329–344. https://doi.org/10.1002/aja.1001480304.
67. Hermanson, J.W.; Lennard, P.R.; Takamoto, R.L. Morphology and Histochemistry of the Ambiens Muscle of the Red-Eared Turtle (Pseudemys Scripta). J. Morphol. 1986, 187, 39–49. https://doi.org/10.1002/jmor.1051870104.
68. Karlsen, K. The Location of Motor End Plates and the Distribution and Histological Structure of Muscle Spindles in Jaw Muscles of the Rat. Acta Odontol. Scand. 1965, 23, 521–547. https://doi.org/10.3109/00016356509021768.
69. Kubota, K.; Masegi, T. Muscle Spindle Distribution in Snout Musculature of the Japanese Shrew-Mole. Anat. Rec. 1972, 172, 703–709. https://doi.org/10.1002/ar.1091720409.
70. Kubota, K.; Masegi, T. Proprioceptive Innervation of the Masticatory Muscles in Pinché. J. Dent. Res. 1975, 54, 788–796. https://doi.org/10.1177/00220345750540041601.
71. Kubota, K.; Masegi, T.; Osanai, K. Muscle Spindle Distribution in the Masticatory Muscle of the Squirrel Monkey (Saimiri Sciurea). Bull. Tokyo Med. Dent. Univ. 1973, 20, 275–286.
72. Kubota, K.; Masegi, T.; Quanbunchan, K. Muscle Spindle Distribution in the Masticatory Muscle of the Tree Shrew. J. Dent. Res. 1974, 53, 538–546. https://doi.org/10.1177/00220345740530030501.
73. Lennartsson, B. Number and Distribution of Muscle Spindles in the Masticatory Muscles of the Rat. J. Anat. 1980, 130 Pt 2, 279–288.
74. Lukas, J.R.; Aigner, M.; Blumer, R.; Heinzl, H.; Mayr, R. Number and Distribution of Neuromuscular Spindles in Human Extraocular Muscles. Investig. Ophthalmol. Vis. Sci. 1994, 35, 4317–4327.
75. Maass, S.; Baumann, K.I.; Halata, Z. Topography of Muscle Spindles and Golgi Tendon Organs in Shoulder Muscles of "Monodelphis Domestica". Ann. Anat. Anat. Anz. Off. Organ. Anat. Ges. 2001, 183, 237–242. https://doi.org/10.1016/S0940-9602(01)80224-7.
76. Maier, A. Occurrence and Distribution of Muscle Spindles in Masticatory and Suprahyoid Muscles of the Rat. Am. J. Anat. 1979, 155, 483–505. https://doi.org/10.1002/aja.1001550406.

77. Maier, A.; Simpson, D.R.; Edgerton, V.R. Histological and Histochemical Comparisons of Muscle Spindles in Three Hind Limb Muscles of the Guinea Pig. J. Morphol. 1976, 148, 185–192. https://doi.org/10.1002/jmor.1051480205.
78. Matsumura, A.; Saito, K. Distribution of Muscle Spindles in the Extensor Digitorum and Hallucis Brevis Muscles of the Macaque as Determined by Plastination. Acta Anat. 1997, 158, 59–67. https://doi.org/10.1159/000147912.
79. Odagiri, N.; Kubota, K.; Shibanai, S. Density of Muscle Spindles in the Jaw Muscles of the Japanese Flying Squirrel and the Guinea Pig. Ann. Anat. Anat. Anz. Off. Organ. Anat. Ges. 1993, 175, 263–270. https://doi.org/10.1016/s0940-9602(11)80015-4.
80. Zenker, W.; Sandoz, P.A.; Neuhuber, W. The Distribution of Anterogradely Labeled I--IV Primary Afferents in Histochemically Defined Compartments of the Rats Sternomastoid Muscle. Anat. Embryol. 1988, 177, 235–243. https://doi.org/10.1007/BF00321134.
81. Takahashi, Y.; Ohmichi, Y.; Lee, P.A.L.M.; Naito, M.; Nakano, T.; Kakizaki, H. Muscle Spindles in the Levator Palpebrae Superioris Muscle of Human Adults. J. Craniofacial Surg. 2021, 32, 1532–1534. https://doi.org/10.1097/SCS.0000000000007135.
82. Smith, K.K. Histological Demonstration of Muscle Spindles in the Tongue of the Rat. Arch. Oral. Biol. 1989, 34, 529–534. https://doi.org/10.1016/0003-9969(89)90091-5.
83. Scott, J.J.; Young, H.. The Number and Distribution of Muscle Spindles and Tendon Organs in the Peroneal Muscles of the Cat. J. Anat. 1987, 151, 143–155.
84. Sanders, I.; Han, Y.; Wang, J.; Biller, H. Muscle Spindles Are Concentrated in the Superior Vocalis Subcompartment of the Human Thyroarytenoid Muscle. J. Voice: Off. J. Voice Found. 1998, 12, 7–16. https://doi.org/10.1016/s0892-1997(98)80070-2.
85. Saigusa, H.; Yamashita, K.; Tanuma, K.; Saigusa, M.; Niimi, S. Morphological Studies for Retrusive Movement of the Human Adult Tongue. Clin. Anat. 2004, 17, 93–98. https://doi.org/10.1002/ca.10156.
86. Sahinen, F.M.; Kennedy, W.R. Distribution of Muscle Spindles in the Human First Dorsal Interosseus. Anat. Rec. 1972, 173, 151–155. https://doi.org/10.1002/ar.1091730204.
87. Richmond, F.J.R.; Stuart, D.G. Distribution of Sensory Receptors in the Flexor Carpi Radialis Muscle of the Cat. J. Morphol. 1985, 183, 1–13. https://doi.org/10.1002/jmor.1051830102.
88. Richmond, F.J.R.; Singh, K.; Corneil, B.D. Marked Non-Uniformity of Fiber-Type Composition in the Primate Suboccipital Muscle Obliquus Capitis Inferior. Exp. Brain Res. 1999, 125, 14–18. https://doi.org/10.1007/s002210050652.
89. Duron, B.; Jung-Caillol, M.C.; Marlot, D. Myelinated Nerve Fiber Supply and Muscle Spindles in the Respiratory Muscles of Cat: Quantitative Study. Anat. Embryol. 1978, 152, 171–192. https://doi.org/10.1007/BF00315923.
90. Rokx, J.T.; van Willigen, J.D.; Jansen, H.W. Muscle Fibre Types and Muscle Spindles in the Jaw Musculature of the Rat. Arch. Oral. Biol. 1984, 29, 25–31. https://doi.org/10.1016/0003-9969(84)90038-4.
